# Dietary Supplementation with Inulin Modulates the Gut Microbiota and Improves Insulin Sensitivity in Prediabetes

**DOI:** 10.1155/2021/5579369

**Published:** 2021-06-29

**Authors:** Xiaojing Wang, Tong Wang, Qian Zhang, Li Xu, Xinhua Xiao

**Affiliations:** ^1^Department of Endocrinology, NHC Key Laboratory of Endocrinology, Peking Union Medical College Hospital, Chinese Academy of Medical Sciences and Peking Union Medical College, Beijing, China; ^2^Department of Endocrinology, Beijing Tsinghua Changgung Hospital, Tsinghua University, Beijing, China; ^3^State Key Laboratory of Membrane Biology and Tsinghua-Peking Center for Life Sciences, School of Life Sciences, Tsinghua University, Beijing, China

## Abstract

**Aims:**

Accumulating evidence indicates gut microbiota dysbiosis is involved in metabolic disorders, including prediabetes. The prebiotic inulin has been frequently reported to exert beneficial effects on the host metabolism. Here, we aimed to evaluate whether dietary supplementation with inulin modulates gut microbiota structure in prediabetes, affecting glucose and lipid metabolism.

**Methods:**

We performed a prospective single-arm study. A total of 49 subjects with prediabetes (WHO 1999 criteria) were voluntarily enrolled. Each subject received a daily supplement with 15 g of inulin for 6 months. Glucose and lipid metabolic parameters and gut microbiota were analyzed at baseline and at 3 and 6 months after inulin intervention. Intestinal microbiota profile was evaluated using the Illumina MiSeq platform based on V3-V4 bacterial 16S rRNA gene.

**Results:**

The mean age of 49 subjects was 56.6 ± 6.9 years and BMI was 25.07 ± 3.02 kg/m^2^. After 24 weeks of prevention, inulin significantly decreased fasting insulin (2.38 ± 0.50 vs. 2.22 ± 0.62, *P*=0.03) and 2-hour post-OGTT insulin (4.01 ± 0.77 vs. 3.74 ± 0.76, *P*=0.02) and improved HOMA-IR (1.05 ± 0.53 vs. 0.85 ± 0.66, *P*=0.03). Gut microbiota analysis indicated that inulin supplement resulted in an increase in the relative abundance of Actinobacteria, Bifidobacteriales, Bifidobacteriaceae, Lactobacillaceae, *Bifidobacterium*, *Lactobacillus*, and *Anaerostipes* both at 3 and 6 months, while with a decrease in the relative abundance of *Alistipes*. Spearman correlation analysis revealed altered microbial community was associated with glucose and lipids metabolic parameters.

**Conclusions:**

Inulin supplementation improves insulin resistance of prediabetes and exerts beneficial effects on modulating the intestinal microbiota composition. These findings suggest that insulin may be a potentially novel and inexpensive intervention for prediabetes.

## 1. Introduction

Prediabetes represents an intermediate state between normal glucose tolerance and diabetes, which is characterized by impaired fasting glucose (IFG) and/or impaired glucose tolerance (IGT) [1]. These impairments have often been traced back to the emergence of insulin resistance, which commonly precedes the development of diabetes by several years [2, 3]. In China, 50.1% of adults have been found to have prediabetes, and the prevalence of prediabetes was relatively high in wealthy East China village [4]. Despite prediabetes often being asymptomatic, there is a high risk of progression to type 2 diabetes in those patients [5]. Additionally, prediabetic dysglycaemia increases the risk of long-term microvascular and macrovascular complications of diabetes, such as cardiovascular disease and stroke [6, 7].

Evidence has been accumulating that gut microbiota play important roles in health maintenance. The intestinal microbiota, consisting of more than 1000 species, mediates the host metabolism by producing metabolites involved in inflammation and gut barrier integrity [8]. Gut microbial dysbiosis has been reported to be associated with metabolic disorders including type 2 diabetes, obesity, insulin resistance, and prediabetes [9]. One study showed that individuals with prediabetes have aberrant intestinal microbiota characterized by a decreased abundance of the genus *Clostridium* and the mucin-degrading bacterium *A. muciniphila* [10]. Therefore, microbiota-targeted interventions that help restore intestinal microbiome structure have suggested a promising strategy in the prevention of prediabetes.

In recent years, prebiotics have gained the great interest of public and researchers as they beneficially modulate gut microbiota composition [11, 12]. Inulin, a well-known prebiotic, has been frequently reported to exert a large number of beneficial effects on the host metabolism including improving glucose and lipid metabolism and protecting against weight gain in animal and human studies [13, 14]. A RCT study recently showed that probiotics supplementation significantly increased the abundance of the *Bacteroides fragilis* to *Escherichia coli* in the adults with prediabetes [15]. However, the effect of inulin on the intestinal microbiota ecosystem in prediabetic individuals remains unclear.

Thus, in the present study, we focused on prediabetes and aimed to evaluate the effect of dietary supplementation with inulin on gut microbiota structure using a high-throughput sequencing platform. Furthermore, the association between gut microbial changes and host glucose and lipid metabolism indices were also explored.

## 2. Materials and Methods

### 2.1. Subjects

A total of 49 subjects were voluntarily enrolled between July 2016 and December 2017. Each participant met the following inclusion criteria: between 18 and 70 years old, BMI between 18 and 35 kg/m^2^, fasting plasma glucose between 6.1 mmol/L and 7.0 mmol/L ,and/or 2 h postprandial serum glucose levels between 7.8 mmol/L and 11.1 mmol/L following a 2-hour 75 g oral glucose tolerance test (the test was performed after a washout period of 4 weeks). During the washout period, subjects were asked to keep their dietary and physical activity habits. The exclusion criteria were gastrointestinal tract disease, renal and or liver disorder, psychiatric disorders, antibiotic use, probiotic, prebiotic, and symbiotic use during the past 3 months, and or during the intervention period, taking any other drugs that may affect gut microbiota and pregnancy. All participants provided written informed consent. The study was approved by the local Ethics Committee of Peking Union Medical College Hospital (No. S749), and written informed consent was obtained from all participants.

### 2.2. Dietary Intervention

Each subject received a daily supplement with 15 g of inulin for 6 months. The products were kindly supplied by Inuling Biosciences Company, Wuhan, China. The inulin powder was added in warm drinks, and compliance was evaluated by returned sachet counts. The participants were instructed not to change their dietary and physical activity habits, and they were asked to record daily diary during the intervention period to assess dietary intake, exercise, and side effects. Contact with subjects was maintained weekly during the study, and any concerns were addressed.

### 2.3. Biochemical Analysis and Anthropometrics

Height, weight, and blood pressure were measured at baseline and repeated at 12 weeks and 24 weeks during intervention. Liver and renal function, lipid profiles, and glycated hemoglobin (HbA1c) levels were also evaluated at each follow-up visit. Oral glucose tolerance test (OGTT) was performed at the baseline and the end of the treatment (24 weeks). HbA1c was determined using high-performance liquid chromatography (HPLC). Serum insulin and C-peptide were measured by direct chemiluminescence immunoassay (SIMENS ADVIA Centaur XP, Germany). The glucose oxidase method was used to measure the plasma glucose levels. All laboratory tests were performed after an 8-hour overnight fast. Homeostatic model assessments of islet B cell function (HOMA-B) and basal insulin resistance (HOMA-IR) were evaluated. The calculation formulas were as follows: HOMA-B = fasting serum insulin (mU/L) × 20/(fasting plasma glucose in mmol/L-3.5), HOMA-IR = fasting serum insulin (mU/L) × fasting plasma glucose (mmol/L)/22.5.

### 2.4. Gut Microbiota Analysis

Stool samples were collected at the beginning and each follow-up visit (12 weeks and 24 weeks). All participants were provided with FloraPrep^TM^ specimen collection kits for stool collection. Upon collection, the stool samples were immediately stored at −80°C. Microbial DNA was extracted from fecal samples using ZR Fecal DNA MiniPrep (D6010, Zymo Research, America) according to manufacturer's protocols. The V3-V4 hypervariable region of the 16S rRNA bacterial gene was amplified by polymerase chain reaction (PCR) (95°C for 2 min, followed by 15 cycles at 95°C for 30 s, 57°C for 30 s, and 72°C for 30 s, and a final extension at 72°C for 10 min). Amplicons were purified using a quick PCR purification kit (Qiagen, Germany). Purified amplicons were then sequenced by Illumina MiSeq 2 *∗* 300 bp the paired-end sequencing platform (Suzhou Admera Med-tech Co., Ltd).

Raw data were filtered with a specific standard to obtain high-quality clean tags using QIIME (V1.8.0). The final qualified sequences were clustered as operational taxonomic units (OTUs) based on 97% similarity threshold using UPARSE. Representative sequences for each OTU were annotated with taxonomic information based on the RDP classifier version 2.2 algorithm by aligning against the GreenGene database. Alpha-diversity and beta-diversity analyses were carried out using QIIME (V1.8.0). ACE index, Shannon's index, Simpson's index, and Chao1 estimates were evaluated. Permutational multivariate analysis of variance (PERMANOVA) was used to compare the beta-diversity. The correlations between the relative abundance of bacterial taxa and clinical parameter were analyzed by using Spearman's correlation.

### 2.5. Statistical Analysis

Data were analyzed using the SPSS statistical software package version 16. Insulin, HOMA-IR, and HOMA-B were logarithmical to normal distribution and expressed as mean ± standard deviation. A one-way repeated-measures analysis of variance with Tukey's post hoc test or paired *t*-test was used for clinical parameters comparisons between baseline and after intervention at each time point. *P* value <0.05 was considered statistically significant.

## 3. Results

### 3.1. Anthropometric and Clinical Parameters

The mean age of 49 subjects was 56.6 ± 6.9 years (37 to 69 years old) and BMI was 25.07 ± 3.02 kg/m^2^. Male subjects accounted for 32.7% (16/49). After 24 weeks of prevention, inulin did not significantly modify HbA1c, 2-hour post-OGTT glycemia, and lipid profiles including total cholesterol, triglycerides, high-density lipoprotein cholesterol (HDL-c), and low-density lipoprotein cholesterol (LDL-c). However, inulin significantly improved fasting insulin, 2-hour post-OGTT insulin, and HOMA-IR. We also observed inulin significantly decreased fasting blood glucose at 12 weeks, but the changes were not persistent. The detailed information is given in Table 1.

### 3.2. Analysis of the Gut Microbiota Composition

**Figure 1:**
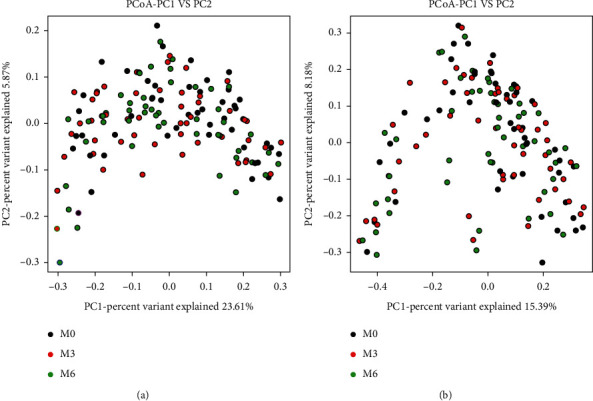
Principal component analysis plot based on Bray-Curtis distance (a) and unweighted UniFrac distances (b) for fecal microbiota. M0, baseline; M3, at three months after inulin intervention; M6, at six months after inulin intervention.

To explore the effect of inulin on gut microbiota structure, we conducted 16s rRNA gene sequencing using fecal samples collected at weeks 0, 12, and 24 during inulin intervention. A total of 10138038 high-quality reads were obtained from 147 samples. The alpha-diversity of the gut microbiota community indicated that inulin significantly decreased the ACE richness estimates and the Shannon diversity index at the 24 weeks (Table 2). To compare the beta-diversity of intestinal microbiota before and after treatment, the PCoA analysis based on Bray-Curtis distance and unweighted UniFrac distances was carried out, and the results revealed no statistically significant differences between baseline and at each time (*P* > 0.05) (Figure 1).

**Figure 2:**
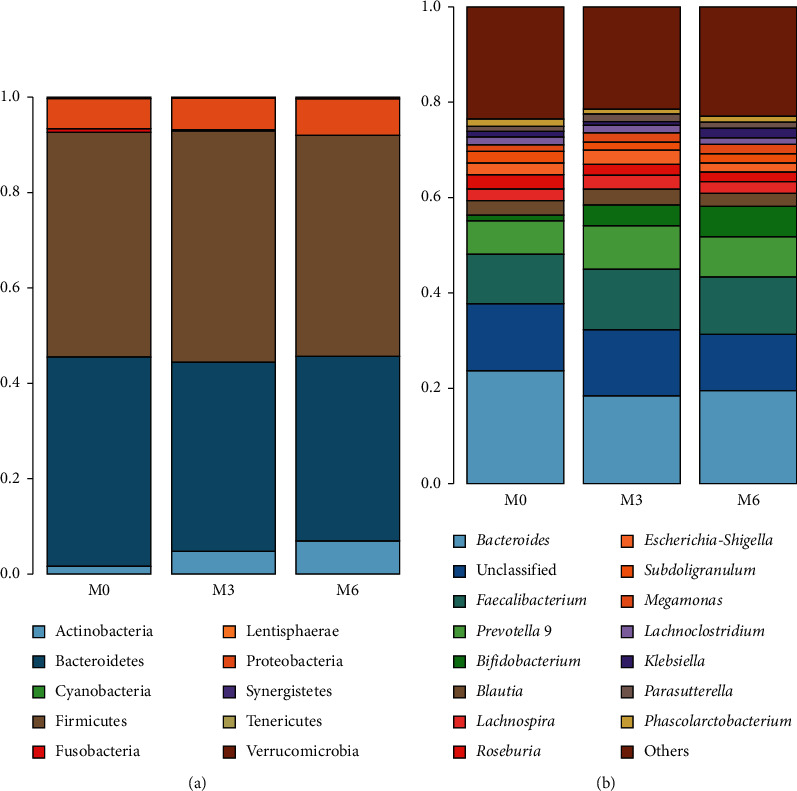
The relative abundance of bacteria at the phylum level (a) and genus level (b) before and after inulin supplement. M0, baseline; M3, at three months after inulin intervention; M6, at six months after inulin intervention.

**Figure 3:**
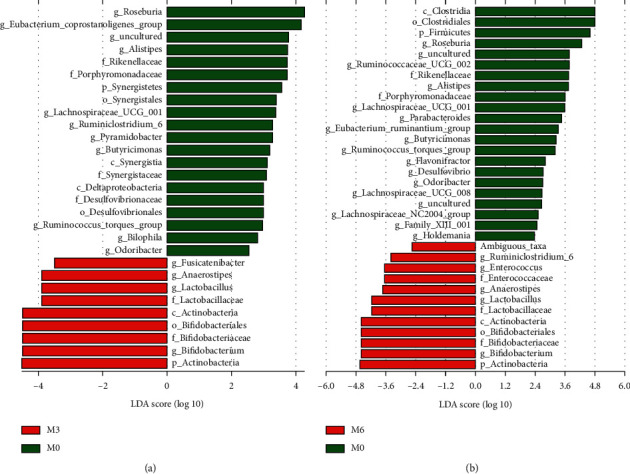
The LEfSe analysis of the gut microbiota from the phylum level down to the genus level. (a) M0 vs. M3; (b) M0 vs. M6. M0, baseline; M3, at three months after inulin intervention; M6, at six months after inulin intervention.

**Figure 4:**
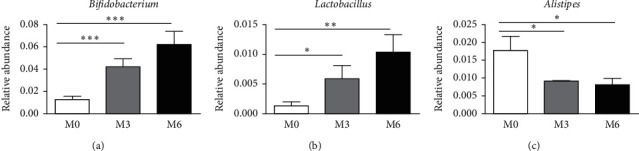
The relative abundance of bacterial taxa at the genus level before and after before and after inulin supplement. (a) *Bifidobacterium*; (b) *Lactobacillus*; (c) *Alistipes* M0, baseline; M3, at three months after inulin intervention; M6, at six months after inulin intervention.

However, inulin supplement significantly altered the composition of the gut microbiota.  Figure 2 shows the relative abundance of intestinal microbiota at the phylum and genus level at each time point. Consistent with the previous reports, the dominant phyla were Bacteroidetes and Firmicutes in all samples. LEfSe analysis indicated that prediabetes patients were dominated with the order Desulfovibrionales, family Desulfovibrionaceae, and genus *Alistipes*. At 3 and 6 months after inulin supplement, the phylum Actinobacteria, the order Bifidobacteriales, family Bifidobacteriaceae and Lactobacillaceae, the genus *Bifidobacterium*, *Lactobacillus*, and *Anaerostipes* were enriched (Figure 3). Metastas analysis further verified the results. The abundance of Actinobacteria, Bifidobacteriales, Bifidobacteriaceae, *Bifidobacterium*, Lactobacillaceae, and *Lactobacillus* were significantly increased at 3 and 6 months compared with baseline, while *Alistipes* showed decrease in relative abundance (Figure 4 and Table S1). The other significantly different bacteria from the phylum level down to the genus level based on Metastas analysis are given in Table S1.

### 3.3. Correlation Analysis between the Fecal Microbiota and Glucose and Lipid Metabolic Parameters

**Figure 5:**
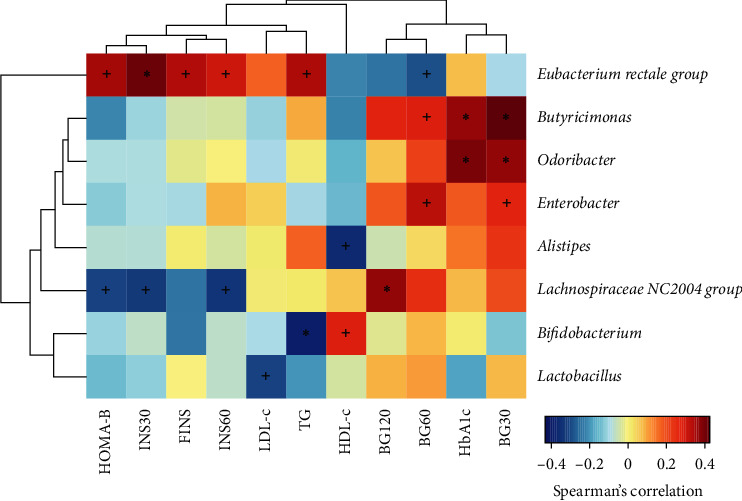
Heat map showing spearman correlations between different gut microbiota at the genus level and glucose and lipid metabolic parameters. ^*∗*^*P* < 0.05, +*q*＜0.05.

To assess whether the distribution of gut microbiota and the glucose and lipid metabolic parameters were correlated, Spearman's correlation analysis was performed. *Eubacterium rectale* group displayed a strong positive correlation with fasting insulin, OGTT-30 min insulin, OGTT-1 h insulin, and triglyceride level. *Butyricimonas* and *Odoribacter* were positively correlated with HbA1c and OGTT 30 min glucose. *Bifidobacterium* showed a negative correlation with HDL-c and positive correlation with triglyceride levels. *Lactobacillus* exhibited a negative correlation with LDL-c, while *Alistipes* were negatively correlated with HDL-c (Figure 5).

## 4. Discussion

The present study explored the role of inulin on gut microbiome in prediabetes patients. The inulin's efficacy on glucose and lipid metabolism were also evaluated. Following 6 months intervention, inulin significantly ameliorated insulin resistance. This is consistent with a previous randomized crossover trial in which inulin was associated with a significant reduction in HOMA-IR in isolated-impaired fasting glucose subjects [16]. In addition, a meta-analysis showed that inulin-type carbohydrates could improve insulin resistant in T2DM, especially in obese T2DM patients [13]. Another meta-analysis included 33 RCTs which further demonstrated that inulin-type fructan (ITF) significantly reduced HOMA-IR in prediabetes or T2DM, as well as in healthy subjects [17].

Inulin, a dietary fiber, cannot be digested and absorbed by human intestine, but can be fermented by the intestinal microbiota, producing a great diversity of metabolites [18]. Inulin has been emerged as important modulators of microbial structure and increasingly investigated their potential benefits for human metabolism through alterations in the intestinal microbiota. Interestingly, the present study showed that inulin supplement significantly decreased the alpha-diversity of gut microbiota. Similarity, another previous study observed that oligofructose-enriched inulin intervention lead to a decrease in alpha-diversity in overweight adults [19]. Moreover, a recent study reported higher consumption of inulin-rich vegetables also decreased gene richness. The relationships between decreased gut microbial richness and health outcomes require further investigation.

An alteration of intestinal microbial composition by inulin in prediabetes was also observed. Inulin significantly increased the abundance of Bifidobacteriales, Bifidobacteriaceae, *Bifidobacterium*, Lactobacillaceae, *Lactobacillus*, and *Anaerostipes*, which were positively associated with lipid metabolic parameters. These results are supported by previous ITF intervention studies. A double-blind randomized cross-over intervention study evaluated the effect of inulin consumption on fecal microbiota composition in healthy adults with midconstipation, and specific inulin-induced changes in relative abundance of *Bifidobacterium* and *Anaerostipes* were identified [20]. Another study also indicated that dietary supplementation with inulin leaded to an increase in *Bifidobacterium faecale*, *Anaerostipes badrus*, and Actinobacteria Class [19]. In addition, a significant bifidogenic effect induced by ITF was observed in obese women [21]. Our previous study also showed that inulin supplement increased the relative abundance of *Bifidobacterium*_*breve* in offspring from high-fat diet- (HFD-) fed dams [22].

*Bifidobacterium* and *Lactobacillus* had been reported to be poorly represented in the fecal samples of diabetes patients and positively impact insulin sensitivity [23, 24]. The two genera were well-known short chain fatty acids (SCFAs) producing bacterium [16]. SCFAs have been shown to stimulate the secretion of glucagon-like peptide-1 (GLP-1) and peptide YY by binding to the G-couple receptors on intestinal L cells and to promote energy expenditure. Additionally, SCFAs have been shown to improve systemic inflammation via reducing intestinal permeability and endotoxaemia, demonstrated to impair insulin signaling and insulin sensitivity [25]. Both animal and human studies have revealed an improved gut barrier function by increasing *Bifidobacterium* abundance [21, 26]. Thus, the beneficial effect of inulin on insulin sensitivity may be partly attributed to the enhanced abundance of SCFAs producing genera.

The present study indicated that inulin supplementation induced a reduction in the abundance of *Alistipes*. *Alistipes* have been shown to be highly abundant in T2DM patients and positively correlate with the inflammatory state, driving insulin resistance [27, 28]. A previous study found that probiotics decreased the abundance of *Alistipes* in mice [29]. HFD intervention was associated with increased *Alistipes* [30]. The sulfate-reducing bacteria of the family Desulfovibrionaceae are key producers of endotoxins. The relative abundance of Desulfovibrionaceae has been reported to be positively associated with plasma lipopolysaccharide levels, which had potent inflammation-inducing capacity and damage gut barrier [31]. Proinflammatory cytokine production was involved in insulin resistance-related metabolic disease. Several studies had demonstrated a marked increase in the abundance of intestinal *Desulfovibrionaceae* both in HFD-induced obesity mice and obesity human subjects [32, 33]. Moreover, *Desulfovibrionaceae* were also enhanced in mice with impaired glucose tolerance [34]. In agreement with these results, our findings showed that the family *Desulfovibrionaceae* dominated in prediabetes before inulin intervention, but not found at 3 and 6 months after inulin supplement. Taken together, the inhibition of potential pathogen-like bacteria and increase in SCFAs producing beneficial bacterium might be responsible alleviating insulin resistance.

There were several limitations in this study. First, the sample size was relatively small. Second, this study was a single-arm, open-label, prepost intervention trial. Lack of an independent control group was another limitation, and potential bias may exist. Third, we were unable to determine the causal relationship between improvements in insulin sensitivity and changes in gut microbiota composition promoted by inulin. The underlying molecular mechanisms warrant further exploration.

In conclusion, the present study indicates that inulin supplementation significantly improved insulin resistance in prediabetes. Our data also showed that inulin was a unique gut microbiota modulating agent that enriched the beneficial bacteria including *Bifidobacterium* and *Lactobacillus* and inhibited the potential pathogen-like bacteria, such as *Alistipes* and *Desulfovibrionaceae*. The inulin-induced metabolic response is possibly associated with the changes of gut microbiota composition. These findings suggest that inulin may be a potentially novel and inexpensive intervention for prediabetes. However, owing to the small sample size and absence of an independent control group of this study, larger scale and randomized controlled trials are therefore warranted to further confirm our findings.

## Figures and Tables

**Table 1:** Comparison with glucose and lipid metabolism parameters before and after inulin supplementation.

	M0	M3	M6	P1	P2
FBG (mmol/L)	5.98 ± 0.71	5.69 ± 0.64	5.78 ± 0.76	**0.01**	0.11
2-hour PBG (mmol/L)	8.13 ± 1.88	8.22 ± 2.19	7.97 ± 2.30	1	1
HbA1c (%)	5.73 ± 0.06	5.76 ± 0.56	5.78 ± 0.08	1	0.63
TG (mmol/L)	1.37 ± 0.10	1.29 ± 0.08	1.29 ± 0.11	0.62	0.37
HDL-c (mmol/L)	1.38 ± 0.04	1.41 ± 0.05	1.37 ± 0.05	1	1
TC (mmol/L)	4.93 ± 1.06	4.78 ± 0.98	4.69 ± 0.99	0.62	0.37
LDL-c (mmol/L)	3.08 ± 0.10	3.03 ± 0.13	3.09 ± 0.85	1	1
FINS (mU/L)	2.38 ± 0.50	—	2.22 ± 0.62	—	**0.03**
PINS (mU/L)	4.01 ± 0.77	—	3.74 ± 0.76	—	**0.02**
HOMA-IR	1.05 ± 0.53	—	0.85 ± 0.66	—	**0.03**
HOMA-B	4.51 ± 0.58	—	4.46 ± 0.66	—	0.37

Data are presented as mean ± SD. FBG, fasting blood glucose; 2PBG, 2-hour postload plasma glucose; HbA1c, glycated hemoglobin; TG, triacylglycerol; HDL-c, high-density lipoproteins; TC, total cholesterol; LDL-c, low-density lipoproteins; FINS, fasting plasma insulin; PINS, 2-hour postload plasma insulin. P1 : M0 vs. M3; P2 : M0 vs. M6. *P* < 0.05 is highlighted in bold. M0, baseline; M3, at three months after inulin intervention; M6, at six months after inulin intervention.

**Table 2:** Alpha-diversity before and after inulin supplementation.

	M0	M3	M6	P1	P2
ACE	238.61 ± 66.10	224.12 ± 58.49	220.59 ± 60.21	0.20	**0.04**
Chao1	243.13 ± 67.43	227.69 ± 60.65	227.05 ± 58.49	0.16	0.08
Shannon	4.89 ± 0.80	4.64 ± 0.77	4.67 ± 0.69	0.10	**0.04**
Simpson	0.92 ± 0.05	0.90 ± 0.05	0.91 ± 0.05	0.16	0.47

Data are presented as mean ± SD. P1 : M0 vs. M3; P2 : M0 vs. M6. *P* < 0.05 is highlighted in bold. M0, baseline; M3, at three months after inulin intervention; M6, at six months after inulin intervention.

## Data Availability

The data used to support the findings of this study are available from the corresponding author upon request.

## Supplementary Materials

Supplementary Materials Supplementary Table S1. The significantly different bacteria from the phylum level down to the genus level based on Metastas analysis.
