# Supplementary material for: Dietary Supplementation with Inulin Modulates the Gut Microbiota and Improves Insulin Sensitivity in Prediabetes
Source: Int J Endocrinol. 2021 Jun 29;2021:5579369. doi: 10.1155/2021/5579369 (PMC8261184; doi:10.1155/2021/5579369)
Supplement: Supplementary Materials — Supplementary Table S1. The significantly different bacteria from the phylum level down to the genus level based on Metastas analysis. [file 5579369.f1.pdf]

Table S1 The significantly different bacteria from the phylum level down to the genus level based on Metastas analysis

	level	M0	M3	M6	P1	Q1	P2	Q2
Actinobacteria	phylum	0.015221	0.045969	0.067709	**0.000999**	**0.002396**	**0.000999**	**0.011858**
Synergistetes	phylum	0.00011	3.41E-05	3.92E-06	0.353646	0.202482	**0.042957**	0.254937
Bacilli	Class	0.003784	0.009223	0.017931	**0.02997**	0.095851	**0.000999**	**0.005354**
Bifidobacteriales	order	0.012545	0.042229	0.061874	**0.000999**	**0.005777**	**0.000999**	**0.005758**
Lactobacillales	order	0.003784	0.009223	0.017931	**0.042957**	0.149037	**0.002997**	**0.011516**
Bifidobacteriaceae	family	0.012545	0.042229	0.061874	**0.000999**	**0.012968**	**0.000999**	**0.003486**
Lactobacillaceae	family	0.001309	0.005861	0.010409	**0.032967**	0.29455	**0.000999**	**0.003486**
Rikenellaceae	family	0.01786	0.00939	0.008241	**0.048951**	0.29455	**0.014985**	**0.035237**
Porphyromonadaceae	family	0.020107	0.011293	0.012134	**0.041958**	0.29455	0.092907	0.104301
Alistipes	genus	0.017758	0.009224	0.00818	**0.03996**	0.295253	**0.018981**	0.110781
Bifidobacterium	genus	0.012545	0.042229	0.061874	**0.000999**	**0.024604**	**0.000999**	**0.012633**
Butyrivibrio	genus	0.000115	0	0	**0.000999**	**0.024604**	**0.000999**	**0.012633**
Eubacterium coprostanoligenes group	genus	0.007805	0.004173	0.005332	**0.021978**	0.240576	0.305694	0.364776
Eubacterium ruminantium group	genus	0.002463	0.000553	0.000454	**0.015984**	0.224954	**0.007992**	0.060638
Eubacterium xylanophilum group	genus	0.000942	0.000251	0.000227	**0.011988**	0.196835	**0.015984**	0.101063
Family XIII UCG-001	genus	0.000166	6.94E-05	8.34E-05	**0.020979**	0.240576	0.072927	0.193414
Lachnospiraceae NK4A136 group	genus	0.004543	0.002104	0.003103	**0.007992**	0.157468	0.27972	0.359717
Lactobacillus	genus	0.001309	0.005861	0.010409	**0.037962**	0.295253	**0.002997**	**0.032485**
Odoribacter	genus	0.000631	0.000367	0.000345	**0.033966**	0.295253	**0.021978**	0.11117
Roseburia	genus	0.049279	0.035722	0.031952	**0.041958**	0.295253	**0.007992**	0.060638
Anaerostipes	genus	0.005071	0.010983	0.011195	0.083916	0.46475	**0.033966**	0.135638
Butyricimonas	genus	0.001245	0.000777	0.000688	0.110889	0.519768	**0.044955**	0.170545
Enterobacter	genus	0.000126	4.82E-05	0.003001	0.332667	0.662678	**0.028971**	0.129302
Eubacterium rectale group	genus	0.002965	0.001286	0.000718	0.156843	0.519768	**0.021978**	0.11117

Lachnospiraceae NC2004 group	genus	0.000144	6.77E-05	2.62E-05	0.272727	0.662678	**0.015984**	0.101063
Pyramidobacter	genus	0.00011	3.41E-05	3.92E-06	0.364635	0.662678	**0.028971**	0.129302
Veillonella	genus	0.001442	0.0048	0.010429	0.338661	0.662678	**0.04995**	0.176861

P1 and Q1: M0 vs M3; P2 and Q2: M0 vs M6. P < 0.05 and Q value < 0.05 are highlighted in bold. M0: baseline; M3: at three month after inulin intervention; M6: at six month after inulin intervention.
